# Molecular methods applied to gynecological diseases: a laboratory review

**DOI:** 10.1590/1806-9282.20241893

**Published:** 2025-06-16

**Authors:** Kátia Candido Carvalho, Giovanna dos Santos Cavalcanti, Grazielle Marques Duarte Purcino, Tatielly Teles de Miranda, Ana Carolina da Costa Sousa, Giovanna Ney Quevedo, Laura Gonzalez dos Anjos, José Maria Soares, Edmund Chada Baracat

**Affiliations:** 1 Universidade de São Paulo, Faculty of Medicine, Laboratory of Structural and Molecular Gynecology (LIM-58) – São Paulo (SP), Brazil.; 2 Bauru Faculty of Medicine, Universidade de São Paulo – São Paulo (SP), Brazil.; 3 Clinical Hospital, Faculty of Medicine, Universidade de São Paulo – São Paulo (SP), Brazil.

## INTRODUCTION

**Figure 1:**
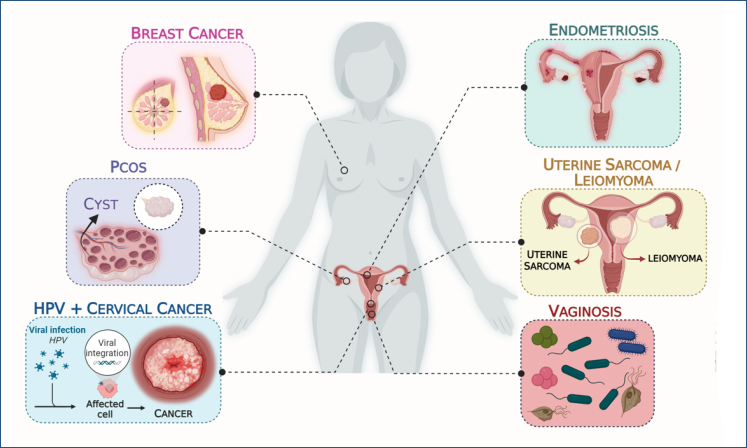
Graphical representation of the most common gynecological diseases covered in the present study. The figure was developed by the authors using Biorender software.

Gynecology is a medical specialty focused on the health of the female reproductive system^1^. Gynecological diseases can affect both the internal and external parts of the genital organs, with causes that include inflammatory conditions (such as endometriosis and pelvic inflammatory disease), infectious diseases like bacterial vaginosis (BV) and chlamydia (caused by bacteria, fungi, and viruses), hormonal issues (such as polycystic ovary syndrome [PCOS]), and genetic factors (such as gynecological cancers, which are multifactorial but have a genetic origin)^1,2^. Figure 1 presents the most common gynecological conditions that were covered in the present review.

Research in molecular biology has highlighted the significance of molecular tests in gynecology and obstetrics, yielding promising results^2^. Techniques such as polymerase chain reaction (PCR) are essential for detecting specific DNA or RNA sequences^1,2^. Next-generation sequencing (NGS) provides comprehensive genomic profiles, revealing genetic variations linked to diseases like gynecological cancer. Immunohistochemistry (IHC) has emerged as a valuable tool for visualizing specific proteins in tissue samples and standardizing molecular markers in cervical cancer^2^.

## MOST COMMON GYNECOLOGICAL DISEASES

### Vaginosis

BV is characterized by an imbalance in vaginal flora, specifically a decrease in beneficial lactobacilli and an increase in anaerobic bacteria. It is the most common cause of discharge, itching, and elevated vaginal pH in women of childbearing age. *Gardnerella vaginalis*, the primary bacterium linked to BV, contributes to this imbalance by producing substances that promote the growth of other anaerobic bacteria, leading to symptoms such as gray discharge with a "fishy" odor^3^.

For the molecular diagnosis of BV, several tests are commonly used. PCR detects the DNA of bacteria associated with BV, while DNA sequencing analyzes the complete genetic material of vaginal bacteria. Nucleic acid hybridization employs labeled DNA/RNA probes to identify specific target bacteria. Additionally, nucleic acid amplification tests (NAATs) can amplify and detect specific nucleic acids, helping differentiate BV from other vaginal infections^3^.

### Polycystic ovary syndrome

PCOS is a common endocrine disorder affecting 5–18% of women of reproductive age. It is characterized by endocrine dysfunction, reproductive issues, hyperandrogenism, oligo- or anovulation, and the presence of polycystic ovaries on ultrasound, following the exclusion of other endocrine disorders^1,4^.

The exact cause of PCOS remains unclear, but some genetic factors may play a role. In addition, microRNAs (miRNAs) have emerged as potential genetic biomarkers due to their regulatory functions and ease of detection^5^.

In molecular biology, the analysis of gene expression through quantitative real-time PCR (qRT-PCR) and IHC has shed light on how certain genes behave in relation to PCOS and its development. Inhibiting the Notch gene signaling pathway by knocking down *NOTCH1* and *RBPJ* genes resulted in a darkening of white adipose tissue (WAT), which improved insulin sensitivity and glucose tolerance. The Notch pathway inhibition using the drug dibenzazepine enhanced Ucp1 expression, suggesting that this signaling pathway may serve as a potential therapeutic target for addressing some consequences of PCOS^4^.

The genes *ACACA*, *SREBP1*, *CPT1*, and *CD36* were also analyzed using qRT-PCR^6^.

### Endometriosis

Endometriosis is a benign gynecological condition affecting 10–15% of women of childbearing age and 3–5% of postmenopausal women^1^. It is characterized by the presence of endometrial-like tissue outside the uterine cavity, leading to chronic inflammation that can impact pelvic tissues and organs^1,7^. This complex condition involves genetic and hormonal factors^7^.

There are three morphological classifications of endometriosis:

1. Peritoneal/superficial: This is the mildest form, affecting the abdominal walls and pelvic organs, with limited tissue penetration.
2. Deep infiltrative: This involves deeper tissue invasion (greater than 5 mm), often affecting the bladder and rectum, causing significant pain and potential organ damage.
3. Ovarian endometriomas: These are cysts formed by endometrial tissue that develop outside the uterus, usually in the ovaries.

The symptoms are multifactorial and may include painful periods, painful intercourse, chronic pelvic pain, and infertility due to the backflow of endometrial tissue during menstruation.

Recent studies using genetic analysis by PCR can help identify changes in some genes related to growth and immunological responses such as the PI3K, AKT, NLRP3, Caspase-1, GSDMD, and GSDMD-N genes, which demonstrate that they may be associated with inflammation and endometriosis while undergoing changes in their signaling pathway^1,7^.

### Breast cancer

Breast cancer is frequently diagnosed in women and continues to see a rise in global incidence^8^. A combination of genetic and non-genetic factors contributes to its onset, including age, reproductive risk factors, exogenous female hormones, lifestyle elements, radiation exposure, mammographic density, and histologic lesions^8,9^. Genetic mutations play a crucial role in the development and progression of breast cancer. Mutations in specific genes, such as *BRCA1* and *BRCA2*, are strongly linked to an increased risk of developing breast cancer, as these genes are involved in DNA repair and maintaining genomic stability^9^. When these genes are mutated, the ability of cells to repair damaged DNA is compromised, leading to the accumulation of genetic alterations that drive cancer development. Additionally, mutations in other genes, such as *TP53*, *PIK3CA*, and *HER2*, can also contribute to breast cancer by affecting key pathways involved in cell growth, division, and survival. Understanding these genetic mutations is essential for identifying individuals at high risk, as well as for the development of targeted therapies that can specifically address the underlying genetic causes of breast cancer. Approximately 8–10% of breast cancer cases are attributed to hereditary mutations, half of which are related to *BRCA1* and *BRCA2* ^8,9^.

### Human papillomavirus+cervical cancer

Human papillomavirus (HPV) causes a widespread sexually transmitted infection that can be contracted through both penetrative and non-penetrative genital skin-to-skin contact. The virus invades basal epithelial cells of mucocutaneous membranes, leading to a range of outcomes, from benign lesions to cancers, including oropharyngeal, cervical, vulvar, vaginal, and penile cancers. Cervical cancer, the fourth most common cancer among women globally, is strongly linked to HPV^10^.

The increased microbial diversity is correlated with HPV expression, as it contributes to increased cervical inflammation and vaginal bacterial infections, thereby favoring the development of precancerous lesions^9^. Additionally, the reduction of lactobacilli is associated with a higher prevalence of the oncogenic proteins E6 and E7 of HPV, which inhibit tumor suppressor genes. Of the more than 20 HPV types affecting the genital epithelium, types 16 and 18 are most commonly associated with cervical cancer^10^.

Various screening methods, including HPV DNA testing, precision tests, viral load quantification, and protein detection via IHC and Western blot, offer specificity and predictive advantages^11^.

### Uterine sarcomas

Uterine sarcomas are a rare form of malignant neoplasm, comprising 3–7% of all uterine tumors and less than 1% of all gynecological cancers, with an annual incidence of approximately 0.30/100,000 women^12^. These tumors often originate in soft tissues, particularly the smooth muscle of the myometrium, with leiomyosarcoma (LMS) being the most common type, typically affecting women over 45 years old^12^. Due to their aggressive nature, the survival rate remains low, even with early diagnosis^12^.

Adenosarcoma, comprising a benign epithelial component and a malignant stromal component, accounts for 5% of uterine sarcomas. It has a lower malignant potential but can still present challenges, including high rates of local recurrence. Uterine sarcomas are histologically classified into carcinosarcomas (50%), endometrial stromal sarcomas (15%), and mixed epithelial/mesenchymal tumors due to their high malignancy^12,13^.

### Leiomyoma

Leiomyomas (uLMs) are benign tumors that originate in the myometrium from smooth muscle cells of the uterus^14^. The causes of their development are not fully understood, but estrogen and hormonal contraceptives may promote their growth^15^. Affecting approximately 70% of women of reproductive age, uLMs are the most common benign genital tumors and typically regress after menopause^14,15^.

The classification of uLMs is based on their location:

- Subserosal: Outer surface of the uterus; may be pedunculated or intraligamentary;
- Submucosal: Located beneath the endometrium, potentially causing complications during pregnancy;
- Cervical: Uterine cervix;
- Intramural: Within the uterine wall, they can grow large enough to distort the uterine cavity.

The uLMs can develop as solitary tumors or in multiples, varying in size throughout the uterus^1,15,16^. Approximately 30% of women are asymptomatic, while the remaining 70% experience symptoms^16^. Common issues include excessive menstrual bleeding, abdominal pain, chronic constipation, and urinary incontinence^14,16^. These tumors may also affect fertility and increase the risk of complications during pregnancy^15,16^.

The diagnosis of uLMs involves a physical examination and symptom analysis, typically confirmed by ultrasound and imaging tests^1,14,15^. Treatment options mainly include hormone-based medications and hysterectomy. Recent molecular biology studies are exploring methods, such as qPCR. A relevant study, using qPCR, showed that the inactivation of the nuclear factor (NF)-κB signaling pathway, through the *TRIM9* gene, was correlated with uLM cell growth and changes in apoptosis. The authors observed that changes in the cell behavior occur depending on the pathway being inactivated, highlighting its importance in understanding the etiology and offering potential for less invasive diagnostic and treatment approaches, with a focus on genetic factors^14–16^.

## MOLECULAR METHODS APPLIED IN THE DIAGNOSIS AND FOLLOW-UP OF THE GYNECOLOGICAL DISEASES

Methods such as PCR enable the detection of specific pathogens like HPV and *Chlamydia*, aiding in the diagnosis of infections and identifying high-risk HPV strains linked to cervical cancer. DNA sequencing plays a crucial role in identifying genetic mutations, such as those in *BRCA1* and *BRCA2*, which predispose individuals to ovarian and endometrial cancers.

Fluorescence in situ hybridization (FISH) detects chromosomal abnormalities in cancer cells, especially for cervical and endometrial cancers, while DNA microarrays allow large-scale gene expression profiling, which helps classify gynecological cancers and personalize treatment. Additionally, molecular biology tests provide rapid identification of infections like gonorrhea and *Chlamydia*. Finally, IHC and miRNA expression profiling aid in identifying biomarkers that inform cancer prognosis and treatment response.

All these molecular techniques have revolutionized gynecological care, improving diagnostic accuracy and treatment efficacy. However, this discussion will focus on more common, affordable, and accessible methods currently available.

### Immunohistochemistry

The IHC technique is widely used in molecular diagnosis because it allows for the detection of specific antigens in tissues or the identification of infectious agents. This is achieved through the binding of these antigens to monoclonal antibodies, which facilitates the visualization and analysis of cellular structures. In certain cases, IHC is crucial for diagnosing oncological diseases by identifying target molecules^17^.

### Genetic sequencing

Sequencing is a molecular biology technique used to determine the exact order of nucleotides in a given DNA or RNA molecule^1^. It is employed to identify and diagnose genetic diseases, perform gene cloning, conduct phylogenetic studies, and identify microorganisms. Recently, an NGS method has been developed. NGS allows for a higher volume of data analysis in a shorter amount of time. This technique is particularly useful for diagnosing uterine sarcomas, as these tumors are heterogeneous and involve specific genes—often gene fusions—that are linked to tumor phenotypes^1^.

Both methods share similar enzymatic principles, but NGS bypasses the need for separating enzymatic nucleotide incorporation from the sequence ladder and data acquisition. This innovation allows NGS to generate sequence data from tens of thousands to billions of templates simultaneously^1^.

### Polymerase chain reaction methods and gene expression analysis

The PCR has progressed through three generations: conventional PCR, qRT-qPCR, and digital PCR (dPCR)^18,19^. These techniques serve to amplify and analyze DNA, presenting a swift and cost-effective approach recognized for its high sensitivity and accuracy in molecular detection^16–19^. Applications encompass mRNA detection, rare mutation identification, and the quantification of copy number variation^19,20^.

While evaluating HPV, qRT-PCR measures the viral load. Quantifying HPV viral load and detecting integration are proposed as predictive indicators for disease progression and severity^11,19^.

Gene expression analysis is a technique that consists of the quantification of messenger RNA (mRNA) levels and the detection of specific genes^21^. It has a great advantage in discovering the genetic origin of diseases aiming at their development, from the activation of the gene to its phenotype^18,21^.

Table 1 presents a summary of the gynecological diseases discussed, along with the techniques and molecular targets identified to date for diagnosing each condition and their potential therapies.

**Table 1:** Gynecological diseases, techniques applied to their management, and molecular targets for each disease's diagnosis and therapy

Disease	Molecular techniques	Molecular biomarkers	Potential therapeutic targets
Vaginosis	Polymerase Chain Reaction (PCR)	IL1b, IL10, IL18, 16s rRNA, Ape2, Sec21, GATA3, and TLR4	PEV7, NDV-3, Mab-B6.1, PCA-2, Als1, Als3, rHyr1-N (CFA), NDV-3A, and NXT-2
	Immunohistochemistry	IL1b, IL10, IL18, GATA3, and TLR4	
	Sequencing	16s rRNA, IL1b, IL10, IL18, GATA3, and TLR4	
Polycystic Ovary Syndrome (PCOS)	Analysis of Quantitative PCR in Real Time (qRT-PCR)	Corin, ACSS2, LPIN1, NR4A1, CAPN, CAPN2, CAPN10, AMH, ACSL5, NLRP12, CCRL2, miR-4488, JDP2, and HMOX1	ACSS2, LPIN1, NR4A1, CAPN2, ACTB, JUN, PTEN, KRAS, MAPK1, and FDX1
	Immunohistochemistry	Galectina-3, CAPN2, NR4A1, AMH, and HMOX1	
	Sequencing	CAPN10, NLRP12, miR-4488, JDP2, and HMOX1	
Endometriosis	Genetic Analysis by the Polymerase Chain Reaction (PCR)	Frizzled-7, FBXO45, TSC22D4, HOXA10, COX-2, IL-12B, GATA6, miR-30c-5p, miR-146a-5p, CCND2, and NOTCH2	FZD7, BST2, IGF2BP1, METTL3, CBLL1, YTHDF2, CXCL12, ROBO3, and SCG2
Breast cancer	Polymerase Chain Reaction (PCR)	CHEK2, PALB2, BRIP1, RAD50, CDH1, and BRCA1/2	KRT81, HER2/neu-positive, HOTAIR, SPRY4-IT1, GAS5, and PANDAR
	Immunohistochemistry	CHEK2, PALB2, RAD50, CDH1, and BRCA1/2	
	Next-Generation Sequencing (NGS)	CHEK2, PALB2, BRIP1, RAD50, CDH1, and BRCA1/2	
HPV + Cervical cancer	Gene Expression Analysis by Polymerase Chain Reaction (PCR)	16S rRNA, TLR9, FMN2, EDNRB, ZNF671, TBXT, MOS, ACTN1, BMPR2, ELK4, EP300, GNAQ, and PI3K/AKT	onco genes E6 e E7, Gardasil-9, ACT, PD-1/PD-L1, ADXS11-001,HB-201, HB202, Vvax001, BNT113, PVX-2, and PVX-6
	Immunohistochemistry	TLR9, EDNRB, ZNF671, TBXT, MOS, ACTN1, BMPR2, ELK4, EP300, GNAQ, and PI3K/AKT	
	Next-Generation Sequencing (NGS)	TLR9, FMN2, EDNRB, ZNF671, TBXT, MOS, ACTN1, BMPR2, ELK4, EP300, GNAQ, and PI3K/AKT	
Uterine sarcoma	Polymerase Chain Reaction (PCR)	t(7;17) (p15;q21), JAXF1, SUZ12, P53, FGF23, and TSCA	PDGFR, KIT, VEGFR, oncoprotein 14-3-3, and RG7112 (MDM2)
	Immunohistochemistry	P53, Ki67, P16, SMARCA4 (BRG1), HMB-45, MELAN-A, SATB2, FGF23, and TSCA	
	Next-Generation Sequencing (NGS)	t(7;17) (p15;q21), JAXF1, SUZ12, P53, FGF23, and TSCA	
Leiomyoma	Genetic Analysis by the Polymerase Chain Reaction (PCR)	DPP6, MFAP5, MED12m, HMGA2, IFITM1, LRG1, hsa_circ_0083920, and circ_0017248	hsa_circ_0056686, NGF, MAP-2, SYP, CB1, FAAH, and GPR55
	Immunohistochemistry	H-caldesmon, IFITM1, LRG1, and CD56	
	Sequencing	MED12m, HMGA2, DPP6, MFAP5, LRG1, hsa_circ_0083920, and circ_0017248	
